# 3,3′-[(*tert*-Butoxy­carbon­yl)aza­nedi­yl]dipropanoic acid

**DOI:** 10.1107/S1600536809018911

**Published:** 2009-05-29

**Authors:** Yuan Tao, Yu-Feng Liang, Xiao-Qiang Guo, Zhi-Hua Mao, Qing-Rong Qi

**Affiliations:** a Department of Medicinal Chemistry, West China School of Pharmacy, Sichuan University, Chengdu 610041, People’s Republic of China; b Faculty of Biotechnology Industry, Chengdu University, Chengdu 610106, People’s Republic of China; c The Center for Testing and Analysis, Sichuan University, Chengdu 610064, People’s Republic of China

## Abstract

The title compound, C_11_H_19_NO_6_, is an important inter­mediate for the synthesis of cephalosporin derivatives. The N atom is in a planar configuration. In the crystal, mol­ecules are linked into zigzag layers parallel to (100) by O—H⋯O hydrogen bonds.

## Related literature

The condensation of the title compound with cephalosporin may improve the pharmacokinetics, see: Sakagami *et al.* (1990 ▶, 1991 ▶); Uhrich & Frechet (1992 ▶).
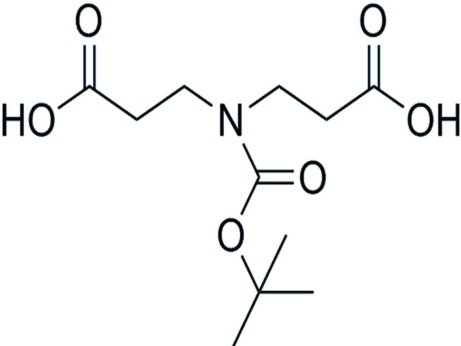


## Experimental

### 

#### Crystal data

C_11_H_19_NO_6_
*M* *_r_* = 261.27
Orthorhombic, 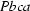

*a* = 10.632 (2) Å
*b* = 14.559 (3) Å
*c* = 18.257 (4) Å
*V* = 2826.1 (11) Å^3^
*Z* = 8
Mo *K*α radiation
μ = 0.10 mm^−1^
*T* = 292 K
0.60 × 0.50 × 0.44 mm

#### Data collection

Enraf–Nonius CAD-4 diffractometer
Absorption correction: none
2979 measured reflections
2601 independent reflections
1050 reflections with *I* > 2σ(*I*)
*R*_int_ = 0.008
3 standard reflections every 200 reflections intensity decay: 1.3%

#### Refinement

*R*[*F* ^2^ > 2σ(*F* ^2^)] = 0.054
*wR*(*F* ^2^) = 0.171
*S* = 1.09
2601 reflections
175 parameters
H atoms treated by a mixture of independent and constrained refinement
Δρ_max_ = 0.22 e Å^−3^
Δρ_min_ = −0.25 e Å^−3^

### 

Data collection: *DIFRAC* (Gabe & White, 1993 ▶); cell refinement: *DIFRAC*; data reduction: *NRCVAX* (Gabe *et al.*, 1989 ▶); program(s) used to solve structure: *SHELXS97* (Sheldrick, 2008 ▶); program(s) used to refine structure: *SHELXL97* (Sheldrick, 2008 ▶); molecular graphics: *ORTEP-3 for Windows* (Farrugia, 1997 ▶); software used to prepare material for publication: *SHELXL97*.

## Supplementary Material

Crystal structure: contains datablocks global, I. DOI: 10.1107/S1600536809018911/ci2799sup1.cif

Structure factors: contains datablocks I. DOI: 10.1107/S1600536809018911/ci2799Isup2.hkl

Additional supplementary materials:  crystallographic information; 3D view; checkCIF report

## Figures and Tables

**Table 1:** Hydrogen-bond geometry (Å, °)

*D*—H⋯*A*	*D*—H	H⋯*A*	*D*⋯*A*	*D*—H⋯*A*
O3—H3*O*⋯O4^i^	0.98 (5)	1.68 (5)	2.653 (4)	174 (4)
O5—H5*O*⋯O2^ii^	0.94 (5)	1.70 (5)	2.628 (3)	168 (4)

Symmetry codes: (i) 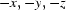; (ii) 
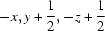
.

**Fig. 1.**
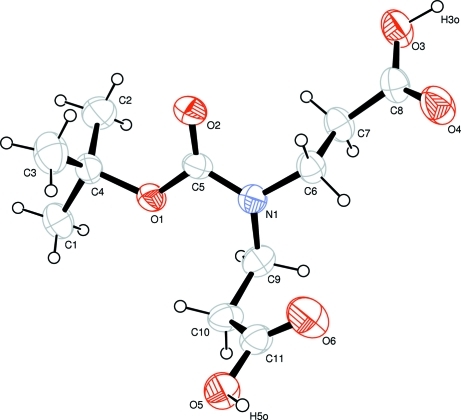
The molecular structure of the title compound, with displacement ellipsoids drawn at the 50% probability level.

**Fig. 2.**
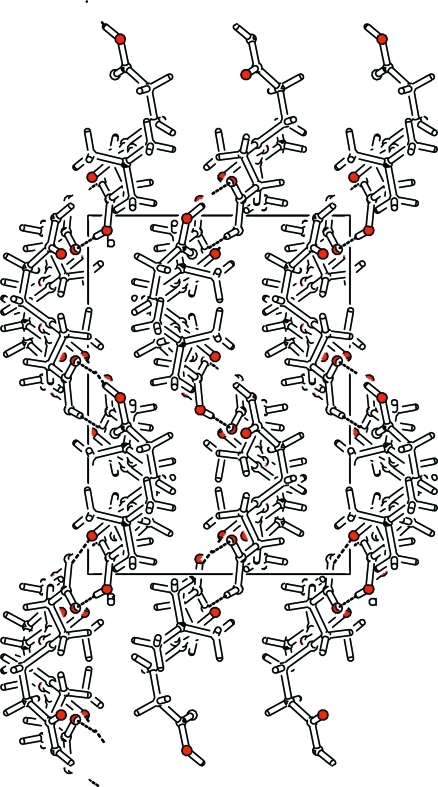
A packing diagram of the title compound. Hydrogen bonds are shown as dashed lines.

## supplementary crystallographic information

### Comment

The title compound is an important intermediate for the synthesis of a new type of cephalosporin. The condensation of the title compound with cephalosporin may improve the pharmacokinetics of the cephalosporin (Sakagami *et al.*, 1990). It has two carboxylic acid functionalities that are available for the condensation with the amino group of cephalosporin, while the protected amine can be easily activated by deprotection, so that it can be condensed with the carboxyl of cephalosporin. The condensation with cephalosporin may increase the drug concentration, control the release of drug and reduce the drug toxicity (Uhrich & Frechet, 1992; Sakagami *et al.*, 1991).

The N atom has a trigonal planar configuration, with sum of bond angles around N1 being 359.8 °. The molecules are linked into zigzag layers parallel to the (100) by O—H···O hydrogen bonds.

### Experimental

Dimethyl 3,3'-azanediyldipropanoate (5.67g, 30 mol) was treated with NaOH solution (4.0g NaOH in 20 ml H_2_O) and stirred at room temperature for 2 h. Then a solution of (Boc)_2_O (7.0g, 32mmol) (Boc is tert-butoxycarbonyl) in tertiary butyl alcohol (10 ml) was added dropwise at 283 K.  The contents were stirred for 30 min at room temperature. The reaction mixture was washed with n-pentane (10 ml × 3) and the aqueous layer was adjusted to a pH of 1.0 with hydrochloric acid and extracted with ethyl acetate. The organic layer was dried (MgSO_4_) and evaporated in vacuo and recrystallized in cyclohexane-ethyl acetate to get colourless crystals.

### Refinement

Hydroxyl H atoms were located in a difference map and refined freely. The remaining H atoms were positioned geometrically (C-H = 0.96–0.97 Å) and refined using a riding model, with *U*_iso_(H) = 1.2–1.5*U*_eq_(C).

### Crystal data

**Table:**

C_11_H_19_NO_6_	*F*(000) = 1120
*M**_r_* = 261.27	*D*_x_ = 1.228 Mg m^−^^3^
Orthorhombic, *P**b**c**a*	Mo *K*α radiation, λ = 0.71073 Å
Hall symbol: -P 2ac 2ab	Cell parameters from 20 reflections
*a* = 10.632 (2) Å	θ = 5.7–6.8°
*b* = 14.559 (3) Å	µ = 0.10 mm^−^^1^
*c* = 18.257 (4) Å	*T* = 292 K
*V* = 2826.1 (11) Å^3^	Block, colourless
*Z* = 8	0.60 × 0.50 × 0.44 mm

### Data collection

**Table:**

Enraf–Nonius CAD-4 diffractometer	*R*_int_ = 0.008
Radiation source: fine-focus sealed tube	θ_max_ = 25.5°, θ_min_ = 2.2°
graphite	*h* = −1→12
ω/2–θ scans	*k* = −3→17
2979 measured reflections	*l* = −10→22
2601 independent reflections	3 standard reflections every 200 reflections
1050 reflections with *I* > 2σ(*I*)	intensity decay: 1.3%

### Refinement

**Table:**

Refinement on *F*^2^	Secondary atom site location: difference Fourier map
Least-squares matrix: full	Hydrogen site location: inferred from neighbouring sites
*R*[*F*^2^ > 2σ(*F*^2^)] = 0.054	H atoms treated by a mixture of independent and constrained refinement
*wR*(*F*^2^) = 0.171	*w* = 1/[σ^2^(*F*_o_^2^) + (0.0701*P*)^2^] where *P* = (*F*_o_^2^ + 2*F*_c_^2^)/3
*S* = 1.09	(Δ/σ)_max_ = 0.001
2601 reflections	Δρ_max_ = 0.22 e Å^−^^3^
175 parameters	Δρ_min_ = −0.25 e Å^−^^3^
0 restraints	Extinction correction: *SHELXL97* (Sheldrick, 2008), Fc^*^=kFc[1+0.001xFc^2^λ^3^/sin(2θ)]^-1/4^
Primary atom site location: structure-invariant direct methods	Extinction coefficient: 0.0127 (17)

### Special details

**Table:**

Geometry. All e.s.d.'s (except the e.s.d. in the dihedral angle between two l.s. planes) are estimated using the full covariance matrix. The cell e.s.d.'s are taken into account individually in the estimation of e.s.d.'s in distances, angles and torsion angles; correlations between e.s.d.'s in cell parameters are only used when they are defined by crystal symmetry. An approximate (isotropic) treatment of cell e.s.d.'s is used for estimating e.s.d.'s involving l.s. planes.
Refinement. Refinement of *F*^2^ against ALL reflections. The weighted *R*-factor *wR* and goodness of fit *S* are based on *F*^2^, conventional *R*-factors *R* are based on *F*, with *F* set to zero for negative *F*^2^. The threshold expression of *F*^2^ > σ(*F*^2^) is used only for calculating *R*-factors(gt) *etc*. and is not relevant to the choice of reflections for refinement. *R*-factors based on *F*^2^ are statistically about twice as large as those based on *F*, and *R*- factors based on ALL data will be even larger.

### Fractional atomic coordinates and isotropic or equivalent isotropic displacement parameters (Å^2^)

**Table:**

	*x*	*y*	*z*	*U*_iso_*/*U*_eq_	
O1	0.1690 (2)	0.18631 (13)	0.36711 (10)	0.0709 (7)	
O2	0.0153 (3)	0.10681 (16)	0.31021 (11)	0.0841 (8)	
O3	0.0720 (3)	−0.04162 (19)	0.08342 (15)	0.0995 (10)	
H3O	0.031 (4)	−0.058 (3)	0.037 (3)	0.129 (16)*	
O4	0.0484 (3)	0.09467 (16)	0.03663 (14)	0.1078 (11)	
O5	0.1228 (3)	0.49187 (18)	0.26626 (13)	0.0833 (8)	
H5O	0.066 (5)	0.526 (3)	0.238 (2)	0.137 (18)*	
O6	0.1031 (3)	0.39281 (16)	0.17583 (15)	0.1142 (11)	
N1	0.1433 (3)	0.20065 (16)	0.24663 (13)	0.0669 (8)	
C1	0.2392 (4)	0.1974 (2)	0.48679 (17)	0.0898 (12)	
H1A	0.3223	0.1849	0.4687	0.135*	
H1B	0.2328	0.1772	0.5367	0.135*	
H1C	0.2233	0.2622	0.4843	0.135*	
C2	0.1697 (4)	0.0451 (2)	0.4391 (2)	0.0981 (14)	
H2A	0.1055	0.0146	0.4112	0.147*	
H2B	0.1700	0.0217	0.4882	0.147*	
H2C	0.2501	0.0342	0.4169	0.147*	
C3	0.0128 (4)	0.1701 (3)	0.4649 (2)	0.1067 (14)	
H3A	−0.0026	0.2343	0.4571	0.160*	
H3B	0.0038	0.1560	0.5160	0.160*	
H3C	−0.0467	0.1347	0.4371	0.160*	
C4	0.1436 (4)	0.1469 (2)	0.44047 (16)	0.0686 (10)	
C5	0.1046 (4)	0.1604 (2)	0.30812 (18)	0.0623 (9)	
C6	0.0904 (4)	0.1711 (2)	0.17744 (16)	0.0688 (10)	
H6A	0.1042	0.2185	0.1409	0.083*	
H6B	0.0003	0.1629	0.1829	0.083*	
C7	0.1484 (3)	0.0821 (2)	0.15127 (17)	0.0747 (11)	
H7A	0.2365	0.0922	0.1400	0.090*	
H7B	0.1437	0.0368	0.1902	0.090*	
C8	0.0838 (4)	0.0459 (3)	0.08533 (19)	0.0719 (10)	
C9	0.2554 (4)	0.2605 (2)	0.24626 (18)	0.0762 (10)	
H9A	0.2844	0.2675	0.1962	0.091*	
H9B	0.3220	0.2308	0.2738	0.091*	
C10	0.2318 (4)	0.3548 (2)	0.27853 (18)	0.0762 (11)	
H10A	0.1970	0.3475	0.3273	0.091*	
H10B	0.3116	0.3865	0.2833	0.091*	
C11	0.1451 (4)	0.4125 (2)	0.2344 (2)	0.0732 (11)	

### Atomic displacement parameters (Å^2^)

**Table:**

	*U*^11^	*U*^22^	*U*^33^	*U*^12^	*U*^13^	*U*^23^
O1	0.0897 (19)	0.0642 (13)	0.0588 (12)	−0.0127 (13)	−0.0146 (12)	−0.0004 (10)
O2	0.097 (2)	0.0806 (16)	0.0750 (16)	−0.0276 (16)	−0.0109 (14)	−0.0061 (12)
O3	0.152 (3)	0.0724 (19)	0.0737 (17)	0.0156 (18)	−0.0244 (17)	−0.0096 (14)
O4	0.164 (3)	0.0759 (17)	0.0831 (17)	0.0102 (17)	−0.0439 (18)	−0.0010 (15)
O5	0.094 (2)	0.0750 (17)	0.0803 (16)	0.0181 (15)	−0.0104 (14)	0.0071 (14)
O6	0.153 (3)	0.0836 (18)	0.106 (2)	0.0274 (18)	−0.053 (2)	−0.0026 (16)
N1	0.078 (2)	0.0626 (15)	0.0595 (16)	−0.0032 (16)	−0.0056 (15)	0.0027 (14)
C1	0.094 (3)	0.103 (3)	0.073 (2)	0.004 (2)	−0.017 (2)	−0.012 (2)
C2	0.132 (4)	0.072 (3)	0.090 (3)	0.002 (3)	−0.012 (3)	0.015 (2)
C3	0.091 (4)	0.132 (3)	0.097 (3)	0.009 (3)	0.013 (3)	−0.017 (3)
C4	0.080 (3)	0.069 (2)	0.0572 (18)	0.002 (2)	−0.0009 (18)	−0.0036 (17)
C5	0.066 (3)	0.054 (2)	0.067 (2)	−0.0066 (19)	−0.0100 (19)	−0.0035 (17)
C6	0.080 (3)	0.065 (2)	0.060 (2)	0.011 (2)	−0.0078 (17)	−0.0011 (16)
C7	0.073 (3)	0.082 (2)	0.069 (2)	0.014 (2)	−0.0123 (18)	−0.0100 (18)
C8	0.081 (3)	0.071 (3)	0.064 (2)	0.025 (2)	−0.0029 (19)	−0.009 (2)
C9	0.069 (3)	0.075 (2)	0.084 (2)	0.005 (2)	−0.0019 (19)	0.014 (2)
C10	0.078 (3)	0.064 (2)	0.087 (2)	−0.010 (2)	−0.023 (2)	0.0155 (17)
C11	0.087 (3)	0.062 (2)	0.071 (2)	−0.006 (2)	−0.011 (2)	0.0116 (19)

### Geometric parameters (Å, °)

**Table:**

O1—C5	1.331 (4)	C2—H2B	0.96
O1—C4	1.482 (3)	C2—H2C	0.96
O2—C5	1.229 (4)	C3—C4	1.499 (5)
O3—C8	1.281 (4)	C3—H3A	0.96
O3—H3O	0.98 (5)	C3—H3B	0.96
O4—C8	1.199 (4)	C3—H3C	0.96
O5—C11	1.315 (4)	C6—C7	1.513 (4)
O5—H5O	0.94 (5)	C6—H6A	0.97
O6—C11	1.193 (4)	C6—H6B	0.97
N1—C5	1.331 (4)	C7—C8	1.482 (5)
N1—C6	1.448 (4)	C7—H7A	0.97
N1—C9	1.477 (4)	C7—H7B	0.97
C1—C4	1.514 (5)	C9—C10	1.515 (4)
C1—H1A	0.96	C9—H9A	0.97
C1—H1B	0.96	C9—H9B	0.97
C1—H1C	0.96	C10—C11	1.486 (5)
C2—C4	1.508 (4)	C10—H10A	0.97
C2—H2A	0.96	C10—H10B	0.97
			
C5—O1—C4	121.8 (3)	O1—C5—N1	113.5 (3)
C8—O3—H3O	108 (2)	N1—C6—C7	111.8 (3)
C11—O5—H5O	110 (3)	N1—C6—H6A	109.3
C5—N1—C6	119.0 (3)	C7—C6—H6A	109.3
C5—N1—C9	120.9 (3)	N1—C6—H6B	109.3
C6—N1—C9	119.0 (3)	C7—C6—H6B	109.3
C4—C1—H1A	109.5	H6A—C6—H6B	107.9
C4—C1—H1B	109.5	C8—C7—C6	111.8 (3)
H1A—C1—H1B	109.5	C8—C7—H7A	109.2
C4—C1—H1C	109.5	C6—C7—H7A	109.2
H1A—C1—H1C	109.5	C8—C7—H7B	109.2
H1B—C1—H1C	109.5	C6—C7—H7B	109.2
C4—C2—H2A	109.5	H7A—C7—H7B	107.9
C4—C2—H2B	109.5	O4—C8—O3	122.6 (3)
H2A—C2—H2B	109.5	O4—C8—C7	122.5 (4)
C4—C2—H2C	109.5	O3—C8—C7	114.9 (3)
H2A—C2—H2C	109.5	N1—C9—C10	113.5 (3)
H2B—C2—H2C	109.5	N1—C9—H9A	108.9
C4—C3—H3A	109.5	C10—C9—H9A	108.9
C4—C3—H3B	109.5	N1—C9—H9B	108.9
H3A—C3—H3B	109.5	C10—C9—H9B	108.9
C4—C3—H3C	109.5	H9A—C9—H9B	107.7
H3A—C3—H3C	109.5	C11—C10—C9	113.9 (3)
H3B—C3—H3C	109.5	C11—C10—H10A	108.8
O1—C4—C3	110.5 (3)	C9—C10—H10A	108.8
O1—C4—C2	109.4 (3)	C11—C10—H10B	108.8
C3—C4—C2	113.4 (3)	C9—C10—H10B	108.8
O1—C4—C1	101.2 (3)	H10A—C10—H10B	107.7
C3—C4—C1	110.4 (3)	O6—C11—O5	122.7 (3)
C2—C4—C1	111.3 (3)	O6—C11—C10	125.6 (3)
O2—C5—O1	123.5 (3)	O5—C11—C10	111.6 (3)
O2—C5—N1	123.0 (3)		
			
C5—O1—C4—C3	−63.2 (4)	C9—N1—C6—C7	89.6 (3)
C5—O1—C4—C2	62.3 (4)	N1—C6—C7—C8	173.1 (3)
C5—O1—C4—C1	179.9 (3)	C6—C7—C8—O4	39.8 (5)
C4—O1—C5—O2	4.4 (5)	C6—C7—C8—O3	−142.1 (3)
C4—O1—C5—N1	−177.7 (3)	C5—N1—C9—C10	−76.4 (4)
C6—N1—C5—O2	−8.8 (5)	C6—N1—C9—C10	115.9 (3)
C9—N1—C5—O2	−176.5 (3)	N1—C9—C10—C11	−67.1 (4)
C6—N1—C5—O1	173.3 (3)	C9—C10—C11—O6	−5.7 (6)
C9—N1—C5—O1	5.6 (4)	C9—C10—C11—O5	177.0 (3)
C5—N1—C6—C7	−78.3 (4)		

### Hydrogen-bond geometry (Å, °)

**Table:**

*D*—H···*A*	*D*—H	H···*A*	*D*···*A*	*D*—H···*A*
O3—H3O···O4^i^	0.98 (5)	1.68 (5)	2.653 (4)	174 (4)
O5—H5O···O2^ii^	0.94 (5)	1.70 (5)	2.628 (3)	168 (4)

Symmetry codes: (i) −*x*, −*y*, −*z*; (ii) −*x*, *y*+1/2, −*z*+1/2.
